# Neuroergonomic Assessment of Hot Beverage Preparation and Consumption: An EEG and EDA Study

**DOI:** 10.3389/fnhum.2020.00175

**Published:** 2020-05-15

**Authors:** Amanda Sargent, Jan Watson, Hongjun Ye, Rajneesh Suri, Hasan Ayaz

**Affiliations:** ^1^School of Biomedical Engineering, Science and Health Systems, Drexel University, Philadelphia, PA, United States; ^2^Lebow College of Business, Drexel University, Philadelphia, PA, United States; ^3^Drexel Solutions Institute, Drexel University, Philadelphia, PA, United States; ^4^Department of Psychology, College of Arts and Sciences, Drexel University, Philadelphia, PA, United States; ^5^Department of Family and Community Health, University of Pennsylvania, Philadelphia, PA, United States; ^6^Center for Injury Research and Prevention, Children’s Hospital of Philadelphia, Philadelphia, PA, United States

**Keywords:** neuroergonomics, consumer neuroscience, electroencephalogram (EEG), electrodermal activity (EDA), emotional valence, market research

## Abstract

Neuroergonomics is an emerging field that investigates the human brain about behavioral performance in natural environments and everyday settings. This study investigated the body and brain activity correlates of a typical daily activity, hot beverage preparation, and consumption in a realistic office environment where participants performed natural daily tasks. Using wearable, battery operated and wireless Electroencephalogram (EEG) and Electrodermal activity (EDA) sensors, neural and physiological responses were measured in untethered, freely moving participants who prepared hot beverages using two different machines (a market leader and follower as determined by annual US sales). They later consumed the drinks they had prepared in three blocks. Emotional valence was estimated using frontal asymmetry in EEG alpha band power and emotional arousal was estimated from EDA tonic and phasic activity. Results from 26 participants showed that the market-leading coffee machine was more efficient to use based on self-reports, behavioral performance measures, and there were significant within-subject differences in valence between the two machine use. Moreover, the market leader user interface led to greater self-reported product preference, which was further supported by significant differences in measured arousal and valence (EDA and EEG, respectively) during coffee production and consumption. This is the first study that uses a multimodal and comprehensive assessment of coffee machine use and beverage consumption in a naturalistic work environment. Approaches described in this study can be adapted in the future to other task-specific machine usability and consumer neuroscience studies.

## Introduction

Neuroergonomics is a multi-disciplinary field that aims to enhance technologies and work environments in terms of safety, effectiveness, and functionality (Ayaz and Dehais, 2019). Neuroergonomics research objectives include understanding neural and physiological factors as they contribute to the real-world task performance, training, and acquisition of new skills concerning work and everyday activities (Parasuraman, 2011; Ayaz et al., 2013; McKendrick et al., 2015). While both neuroscience and human factors research has previously avoided using an active behavior for fear of artifacts contaminating the physiological signal of interest, the development of remote, wireless and portable sensors now allow continuous multimodal monitoring of participants in naturalistic settings (McKendrick et al., 2016; Gramann et al., 2017; Zander et al., 2017; Curtin and Ayaz, 2018; Gateau et al., 2018; Pinti et al., 2018; Dehais et al., 2019).

Neuroergonomics research strives to use neuroimaging techniques to evaluate brain responses in ambulatory settings (Makeig et al., 2009; Ayaz et al., 2013; Mehta and Parasuraman, 2013; Gramann et al., 2014, 2017; Curtin and Ayaz, 2018). The capability for researching outside of traditional laboratory settings has opened the door for the implementation of ecologically valid experimental protocols in fields such as consumer neuroscience. Studying consumer behavior in unrealistic laboratory settings often raises questions about the validity of such results (Boksem and Smidts, 2015; Krampe et al., 2018). Furthermore, recent consumer research studies have typically focused on only one type of product or advertisement when assessing consumers’ behavior (Boksem and Smidts, 2015; Telpaz et al., 2015; Krampe et al., 2018). However, products are rarely featured alone for example in a grocery store (Krampe et al., 2018). Despite a need for researching in naturalistic settings almost all neuromarketing studies to date have attempted to elicit emotional and cognitive responses artificially in a lab (Lim, 2018). Being able to study consumers in naturalistic environments could generate a better understanding of consumers’ behavior and preferences.

Applying neuroscience and more specifically using a neuroergonomics approach to understand marketing and shopping environments provides new opportunities to improve our understanding of consumers’ decision making, and their brand preferences (Harris et al., 2018). The use of neuroimaging and physiological responses along with traditional self-reported and behavioral measures is likely to provide rich and high-resolution data that can be helpful in better understanding consumer preferences. It is then not surprising that several researchers believe that neuroimaging technology could address some of the problems that marketers currently face in not being able to convincingly explain the underlying processes leading to consumption of products and services (Ariely and Berns, 2010; Yoon et al., 2012; Smidts et al., 2014). Traditional consumer research tools, such as focus groups and self-reported measures, incorporate various intrinsic methodological limitations. For instance, consumers may not be able to articulate their preferences fully and might use personal, social, or random bias to make their consumption decisions. Further, such self-reported measures are often intrusive, requiring interruption of the natural engagement with the product or demand a recall of the consumption experience, likely to result in erroneous elicitation of the consumption behavior. Therefore the use of objective neural and physiological measures could complement self-reported measures to provide a richer understanding of consumers’ decision making and their preferences (Ariely and Berns, 2010).

A prominent advantage of neuroergonomics is the use of continuous brain and body measures that provide complementary information to traditional self-reported measures and result in a holistic view of consumers’ product interaction (Ayaz and Dehais, 2019). Using a new generation of wearable and portable neuroimaging sensors, brain activity can be monitored and used to analyze how consumers engage with (interact or consume) products in naturalistic settings (Ayaz et al., 2012; Hsu and Yoon, 2015; Soria Morillo et al., 2016; Barnett and Cerf, 2017; Çakir et al., 2018). Data from these sensors provide new perspectives and information about consumption behaviors that were until now not objectively obtainable through conventional consumer research methods (Calvert and Brammer, 2012; Khushaba et al., 2013). Wireless electroencephalography (EEG) is one such sensor that measures brain dynamics *via* voltage changes over the scalp. So far, tethered EEG has been used in numerous studies to measure the relationship between effect, engagement, and brain activation (Avitan et al., 2009; Guixeres et al., 2017; Lin et al., 2018). Diverse EEG signal components have been studied in consumer neuroscience including P300, N200, theta, gamma, beta, delta, alpha, and most frequently frontal asymmetry (Ma et al., 2008; Ohme et al., 2009, 2010; Cook et al., 2011; Vecchiato et al., 2011a,b; Boksem and Smidts, 2015).

Asymmetric frontal brain activity and affective-valence hypothesis are one of the most known biomarkers in the science of emotion (Palmiero and Piccardi, 2017). Over 70 studies have used EEG to detect emotional processes and frontal asymmetry has been used to understand the relationship between emotion or emotion-related constructs (Harmon-Jones and Allen, 1997, 1998; Coan and Allen, 2004; Palmiero and Piccardi, 2017). Davidson et al. (1979) proposed a model where the left frontal cortex is involved in an approach behavior whereas the right is involved in withdrawal behavior (Ohme et al., 2010). Therefore, emotion will be associated with a right or left asymmetry depending on the extent to which it is accompanied by approach or withdrawal behavior (Davidson, 2004). Previous studies have shown that the more positive a participant’s attitude towards the product or advertisement the higher the frontal alpha asymmetry that can then be used as a predictor for an event-related emotional response (Harmon-Jones et al., 2006, 2010; Ohme et al., 2010; Vecchiato et al., 2010, 2011b; Jenke et al., 2014). The commonly used method to interpret frontal asymmetry is to assess the neural activity at the frontal lobes, mainly F3-F4 and F7-F8 channel couples (Vecchiato et al., 2011b; Çakar and Gez, 2018).

The use of electrodermal activity (EDA), also known as the galvanic skin response (GSR) is another widely adopted tool in consumer research (LaBarbera and Tucciarone, 1995; Boucsein, 2012; Topoglu et al., 2020). EDA is used to measure changes in electrical properties of the skin related to the autonomic nervous system activity. The electrical properties are altered by the electrolytes inside sweat secreted by the eccrine sweat glands (Boucsein, 2012). Eccrine sweat glands play a key role in regulating thermoregulation and are activated by the sympathetic activity of the autonomic nervous system (Dawson et al., 2007) which is responsible for the fight-or-flight response. Hence, EDA measures reflect bodily arousal and can be associated with emotional expressions and behaviors in humans (Critchley, 2002; Cowley et al., 2016). However, one key limitation of such a measure is its lack of valence. Specifically, EDA cannot differentiate between positive and negative arousal and needs to be used in conjunction with other measures (Harris et al., 2018). Studies combining measures on brain activity and EDA could provide evidence for a combined multimodal approach to assess decision making, and to detect perceptions of emotion and motivation (Critchley et al., 2000; Nagai et al., 2004; Ohme et al., 2010; Vecchiato et al., 2010; Wong et al., 2011; Holper et al., 2014).

Usability is the extent to which a product can be efficiently used by operators. Testing usability not only determines whether a product interface will be effective but also result in loyal and satisfied users (Hill and Bohil, 2016). Previous usability research has utilized self-report measures designed to assess user’s mental state while completing a task using the product (Flavián et al., 2006; Gregg and Walczak, 2010; Hill and Bohil, 2016). However, subjective and self-reported indicators such as frustration and satisfaction with a product or its interface are difficult to verbalize and can often vary during different stages of product usage. These limitations make it difficult to assess a product’s usability over time (Hill and Bohil, 2016). Hence recent studies have begun to combine self-reported measures with the brain and body measures to provide a more comprehensive assessment of usability and product satisfaction (Bhatt et al., 2019).

For the current study, we assess the effectiveness of single-serve coffee makers in office settings including the consumption of the prepared coffees. Such a self-service consumption context is becoming popular in both the workplace and at home. According to the National Coffee Association, 41% of coffee drinkers owned a single-serve brewing machine in 2018 (Brown, 2018). Self-service machines are becoming more advanced and an optimal user interface can greatly affect consumer preferences and attitudes (Hubert and Kenning, 2008). Though some researchers suggest that consumers value products more when they play an active role in their production (Atakan et al., 2014), others argue otherwise (Xia and Suri, 2013). No study to our knowledge has investigated the link between the preparation of a product, its consumption, and product valuation for hot beverages and will be the focus of this research.

In this neuroergonomic study, we aim to demonstrate the feasibility of brain and body measurements for assessing the effectiveness of an everyday device, a single-serve coffee machine. Specifically, we looked at the brain and body measures during the preparation and consumption of the beverages. Our motivation was to demonstrate the versatility and applicability of the neuroergonomic approach to assess such everyday devices and do so in a natural setting as have been done more recently with complex aviation systems such as air traffic controllers (Ayaz et al., 2012; Harrison et al., 2014) and pilots during the actual flight (Gateau et al., 2018). Using a comprehensive multimodal approach, we examine the impact of engaging in hot beverage preparation and consumption on the affective states of consumers. We investigated the effect of two single-serve coffee machine user interfaces on consumer experience using mobile EEG and EDA. We chose two hot beverage machines—one from a leader and the other from a follower in this industry (approximately 30-fold difference in annual sales). While users prepared and consumed hot coffee in a realistic office setting, they were continuously monitored with wireless brain and body sensors. Acquired data included self-reported measures, EDA based assessment of arousal, and brain dynamics measured using electroencephalography EEG to assess emotional valence. We expected that market leader would have a more efficient user interface, resulting in less mental effort. We also predicted that such an efficient user interface will also lead to greater product preference. We believe that to date, this is the first study to incorporate a neuroergonomics approach to assess the emotional state changes related to a hot beverage preparation and consumption in an ecologically valid office setting.

## Materials and Methods

### Participants

Twenty-six participants (14 females, mean age = 37.9 years, *SD* = ±13.2 years) volunteered for the study. All confirmed that they met the eligibility requirements of being right-handed with vision correctable to 20/20, did not have a history of brain injury or psychological disorder, and were not on medication affecting brain activity. Before the study, all participants signed consent forms approved by the Institutional Review Board of Drexel University.

### Experimental Procedure

The experiment was performed over a 90-min session. Using a within-subjects repeated measures design, participants prepared hot beverages using the two different machines sequentially (leader, follower) in three blocks. All brand name identifiers on the two machines were covered from participants’ views to prevent confounding effects of brand names on the assessment of user interfaces (Leuthesser et al., 2003; Suri et al., 2013).

Participants took part in this study in a behavioral lab that was modified to simulate a real-world office setting. The office space included a cubicle, a breakroom, and a hot beverage making station. Participants wore wireless EEG and EDA devices so they would be able to move freely throughout the office space. After providing informed consent, participants were fitted with a wireless 12 channel EEG headset (OpenBCI Ultracortex Mark IV) to measure brain electrical activity. A wireless EDA sensor (Neulog GSR Logger Sensor NUL-217) was also attached to their left hand to measure arousal.

After the device set up, for each block, participants completed a task battery containing three cognitive tasks. Tasks were incorporated to represent activity commonly performed in a work/office environment. The first task required participants to perform mental computations. Participants were instructed to enter the correct response on the screen after multiplying or adding the numbers as quickly as possible. Participants were asked to compute single-digit numbers (e.g., 3 + 5 or 3 × 5) in the simple block, and double-digit numbers (e.g., 13 + 21 or 12 × 45) in the challenging block to increase the difficulty of the task. Participants were given up to 10 s to respond to each question with a five-second inter-stimulus period. In between simple and challenging blocks, there was a 15 s rest period. The task lasted a total of 3 min.

The next cognitive task was a rapid visual processing task (RVP). Participants were asked to identify a target (single digit: 5, or triple-digit: 2–4–6) from a sequence of digits that were presented serially one at a time. The digits, from 1 to 9, appeared in pseudo-random order on a screen at the rate of 100 digits per minute. When the participant noticed their assigned target, they responded by pressing the enter key as quickly as possible. Participants were given a total of four target sequences to identify, two simple and two challenging. In between simple and challenging blocks, there was a 15 s rest period. The task lasted a total of 3 min.

The Stroop paradigm was used for the final task in this task battery. In this task, participants were presented with color words (i.e., red, blue, green) and asked to identify which of the two conditions the word belonged. For the first condition, *congruent condition*, the word as displayed in the same color as its name denoted (e.g., “blue” displayed in blue color font) and the second condition, *incongruent condition*, where the word was displayed in a different color font than what its name denoted (e.g., “green” displayed in the yellow color font). Participants pressed the assigned keys to indicate their response. Each stimulus appeared on screen for 2 s followed by a 1 s interstimulus period. In between congruent and incongruent blocks, there was a 15 s rest period. The task lasted a total of 4 min.

After completion of each task battery, participants moved from their cubicle to the hot beverage machine station to prepare hot beverages on both the machines (follower machine; leader machine). To avoid order effect, the order of preparation on the machines was counter-balanced. During the first two blocks, an accompanying computer screen provided participants with stepwise instructions on operating each of the machines. To assess the usability of the machines, no instructions were provided during the third block. Participants were given as much time as they needed to complete the beverage preparation process. During each block participants also chose one of the two beverages to drink. During the first block, participants picked the beverage of their choice. In the second block, participants were constrained to consume the beverage from the machine that they did not select during the first block (constrained choice). The final block again allowed participants to pick the beverage of their choice (free choice). The choice of beverages during the second and the third blocks along with self-reported assessments of these beverages was used to assess the likeability of the beverages. Linear mixed models with repeated measures were used for statistical analysis of self-report and behavioral data. Between and within fixed factors for the model were machine type (follower/leader), block (with/without instructions) on operating the machines, and free choice/constrained choice for consumption.

**Figure 1:**
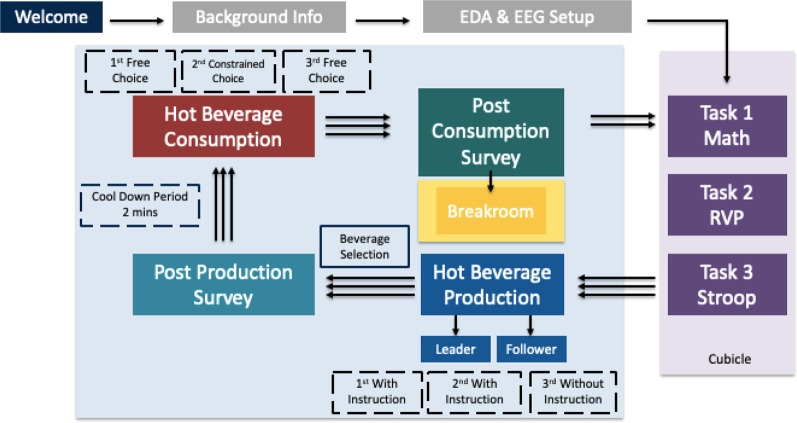
Research procedure.

After preparing the drinks, participants were provided 2-min to allow for the cooling of the beverage before its consumption. During this period, participants were asked to complete a simple non-cognitive task of coloring in the provided coloring books. The cups were weighed before and after drinking to determine the amount of product consumed. Participants were provided the option to add milk and sugar/sweetener to their chosen or assigned beverage and the quantities of each of these additional ingredients were recorded. Participants were given 4 min to consume the beverage. The entire beverage preparation and consumption process took 10 min per block to complete. After each block, participants responded to survey items to assess their mood and attitude towards the machines and the consumed beverage (like/dislike). Finally, participants indicated whether they were interested in returning to work in their cubicle. The experimental design is summarized in Figure 1.

### EDA Acquisition and Analysis

Skin conductance was measured using a wireless EDA sensor (Neulog GSR Logger Sensor NUL-217) in micro Siemens at a sampling rate of 2 Hz *via* Neulog Software (Prasolenko et al., 2015). The EDA used a constant voltage method approach. It included two EDA probes attached using durable rubber-coated wires and two white Velcro finger connectors, placed on the ring and index fingers of the left hand. The sensor is pre-calibrated at the factory. Time synchronized blocks for each block were processed using the MATLAB Toolbox Ledalab (Benedek and Kaernbach, 2010).

For the preprocessing of data, a Butterworth—low pass filter was applied with an order of 2 and a cutoff frequency of 0.1 Hz. After preprocessing the data, continuous decomposition analysis was applied using Ledalab. The data was separated into tonic and phasic activity (Benedek and Kaernbach, 2010). Linear mixed models with repeated measures were used for statistical analysis. Between and within fixed factors for the model were machine type, block with (blocks 1 and 2)/without (block 3) instructions on operating the machines and choice (free choice/constrained).

### EEG Acquisition and Analysis

Raw EEG signals were recorded using a wireless dry electrode EEG system (OpenBCI Ultracortex Mark IV EEG headset). Electrode impedances were reduced to less than 10 kOhms. The EEG channels were positioned according to the 10-20 system at Fp1 Fp2 F7 F8 F3 F4 C3 C4 P7 P8 O1 O2–ground on the right earlobe, reference on the left earlobe. It was sampled at 250 Hz and acquired using OpenViBE Acquisition Server v1.3.0 and OpenVibe Designer v1.3.0. All data were filtered and processed offline after recording.

The recorded EEG signal was preprocessed using the MATLAB toolbox, EEGLAB (Delorme and Makeig, 2004). The signal was high-pass FIR filtered with a filter order of 1,000 at 0.5 Hz and low-pass at 30 Hz. The EEGLAB plugin CleanLine was used to adaptively estimate and remove sinusoidal artifacts from scalp channels using a frequency domain (multi-taper) regression technique with a Thompson F-statistic for identifying significant sinusoidal artifacts. Bad channels were rejected and Artifact Subspace Reconstruction (ASR) was applied to correct continuous data before being re-referenced to average (Mullen et al., 2013). Time synchronized events from the periods of machine interaction (30–60 s) and hot beverage consumption (2 min) were analyzed in the frequency domain where average power spectral density values were calculated for each of the selected intervals. Power spectral density for F7/F8 channel pairs within the alpha frequency band (8–13 Hz) was extracted for each block. A measure of EEG asymmetry was then derived using log-transformed values then subtracting (right-left) to look at the cortical activity. Linear mixed models with repeated measures were used for statistical analysis using NCSS 10 (v10.0.14). Between and within fixed factors for the model were machine type (follower/leader), block (with/without instructions) and choice (free choice/constrained) during consumption, and age. The factor “block” was investigated to determine how the presence of instructions affected usability of both machines. Choice was investigated to determine if participants preferred the beverage of their choosing rather than the constrained choice.

## Results

### Self-reports

#### Self-reports—Ease of Use

**Figure 2:**
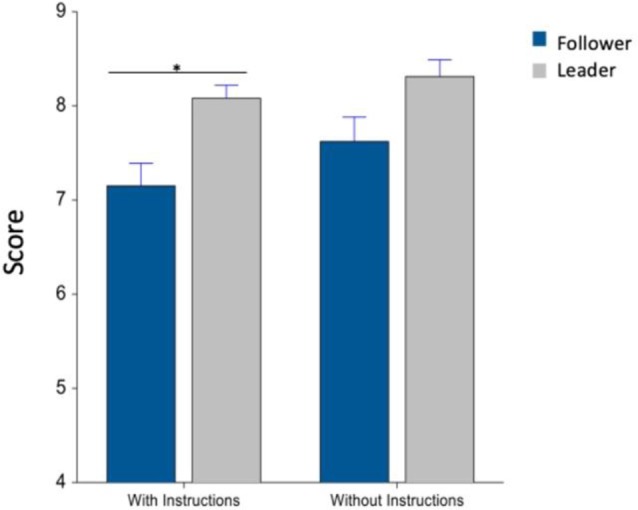
Ease of use of each machine, with and without instructions. Whiskers are standard error of the mean (SEM). *Means statistically significant difference.

After preparing a beverage, participants rated the ease of use of that machine on a 9-point scale (1 = not at all, 9 = extremely). A comparison of self-reported data from both machines shows that there was a significant interaction between machine type and block (*F*_(1,152)_ = 12.9, *p* < 0.001) in Figure 2. Moreover, in *post hoc* results, there was a significant difference between the two machines in the “with-instructions” block (*F*_(1,152)_ = 12.65, *p* < 0.001).

#### Self-reports—Beverage Likeability

**Figure 3:**
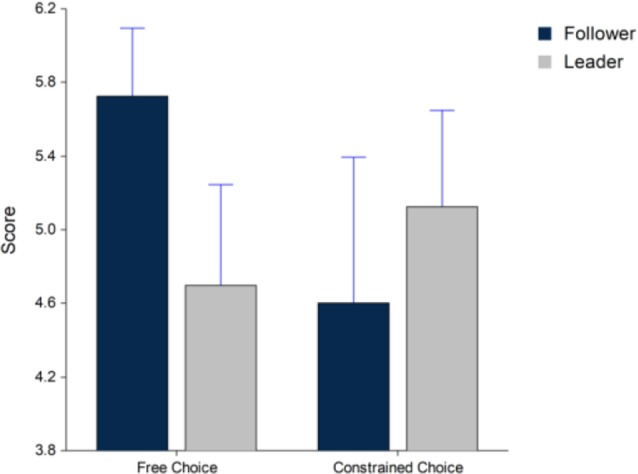
Beverage likeability for the free and constrained choice conditions. Whiskers are SEM.

A comparison of beverage likeability for the beverages prepared on each machine shows that participants liked the beverage prepared on the follower machine better during the free choice condition and leader machine during the constrained choice condition. However, these differences in likeability did not approach significance (*F*_(1,74)_ = 1.92, *p* > 0.05) in Figure 3.

### Behavioral—Drink Preparation and Consumption

**Figure 4:**
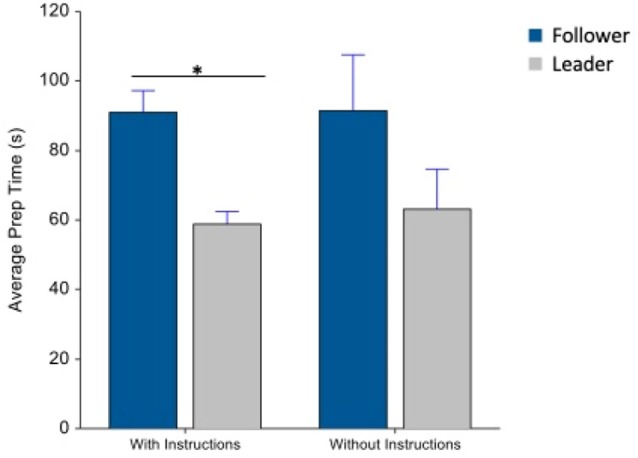
Average preparation time (s), with and without instructions. Whiskers are SEM. *Means statistically significant difference.

Beverage preparation time was also recorded, while participants interacted with the machines. A comparison of preparation time showed a significant main effect of machine type, between follower and leader machines (*F*_(1,62)_ = 12.2, *p* = 0.0009) in Figure 4. *Post hoc* comparisons showed a significant difference between the two machines in the “with-instruction” condition (*F*_(1,62)_ = 10.27, *p* = 0.005).

**Figure 5:**
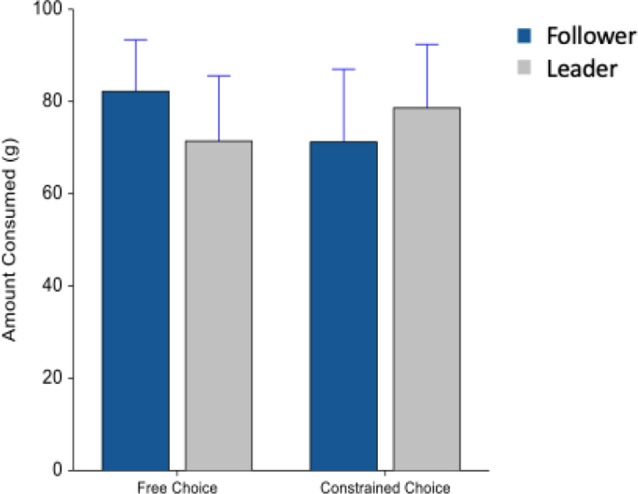
Average beverage consumption (g) during the free and constrained choice conditions. Whiskers are SEM.

After making a beverage on both machines, participants picked one beverage to consume. It was the participant’s choice of beverage in the first block (free choice) but was directed to consume the drink that was not consumed in the second block (constrained choice). There was no significant difference between the volume of beverage consumed during the free and the constrained choice conditions for either machine (*F*_(1,74)_ = 0.37, *p* = 0.545) in Figure 5.

### Electrodermal Activity

**Figure 6:**
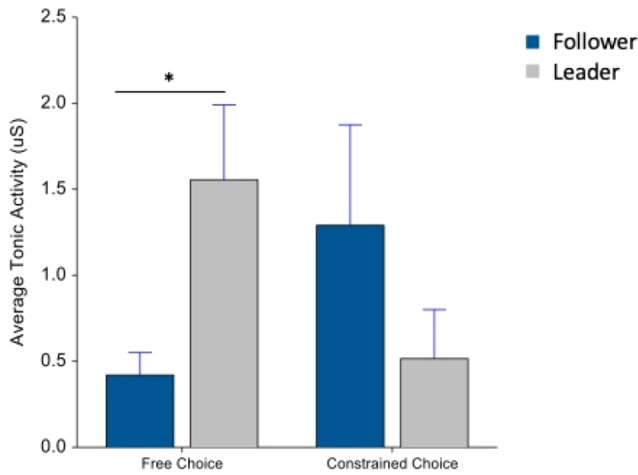
Average tonic activity during beverage consumption. Whiskers are SEM. *Means statistically significant difference.

There was no significant difference in tonic and phasic activity between task conditions and the machines during beverage preparation. In EDA data collected during the beverage consumption period, there was a significant interaction between machine type and choice in tonic activity (*F*_(1,74)_ = 7.09, *p* < 0.01). In a *post hoc* analysis, there was a significant difference between machines during the free choice condition (*F*_(1,74)_ = 7.74, *p* < 0.01) in Figure 6.

**Figure 7:**
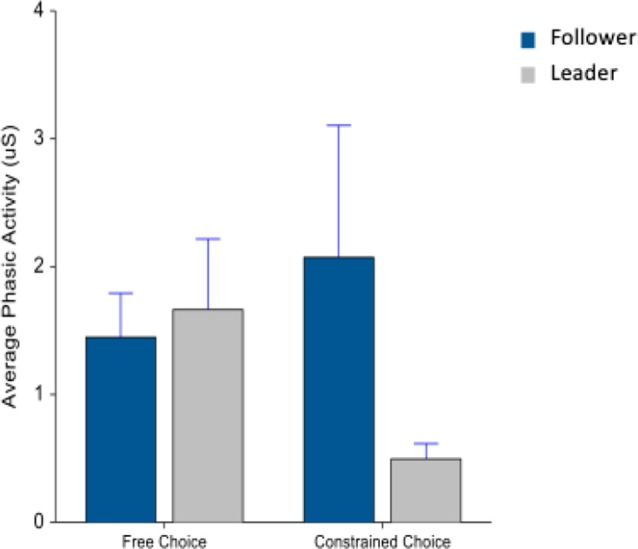
Average phasic activity during beverage consumption. Whiskers are SEM.

Next, looking at the phasic activity during drink consumption, there was a marginally significant interaction between machine type and choice (*F*_(1,74)_ = 2.85, *p* = 0.09) in Figure 7.

### Electroencephalography

**Figure 8:**
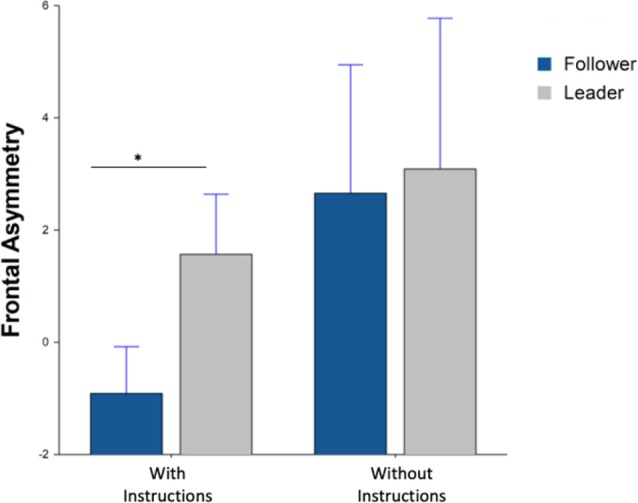
Average frontal asymmetry during beverage preparation. Whiskers are SEM. *Means statistically significant difference.

Alpha frontal asymmetry (F8/F7) during beverage preparation yielded a significant main effect for the block (with/without instructions; *F*_(1,815)_ = 4.72, *p* = 0.03) however there was no significant interaction between the block and machine type (*F*_(1,76.3)_ = 0.8, *p* = 0.37) in Figure 8. *Post hoc* results did indicate a significant difference for the instructions block between leader and follower machine (*F*_(1,78.7)_ = 5.29, *p* = 0.04).

**Figure 9:**
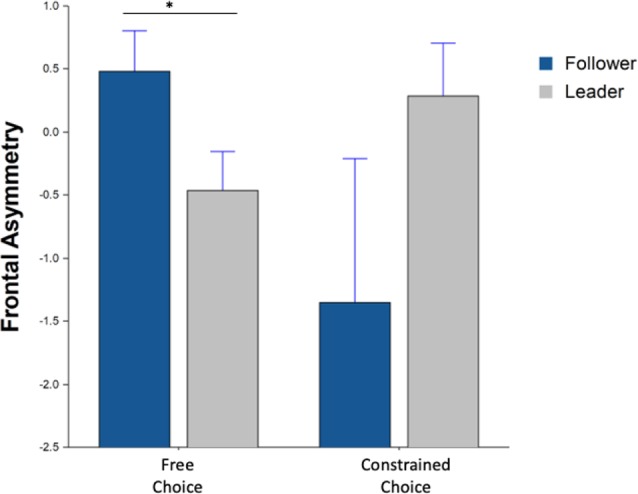
Average frontal asymmetry during beverage consumption Whiskers are SEM. *Means statistically significant difference.

Lastly, the alpha frontal asymmetry of F8/F7 during the beverage consumption period yielded a significant interaction effect between choice and machine type (*F*_(1,25.5)_ = 6.21, *p* = 0.01) in Figure 9. *Post hoc* results further indicated a significant difference for the free choice between leader and follower machine (*F*_(1,39)_ = 6.16, *p* = 0.03).

## Discussion

In this study, we investigated the ease of use of a hot beverage machine and product preference within an ecologically valid office setting. Results from this study suggest that self-reported and behavioral measures along with physiological and neural data (EDA and EEG) provide a multimodal and comprehensive assessment of hot beverage machine use and consumer preferences in a simulated office environment. In an earlier study, we demonstrated the impact of tea and coffee consumption on cognitive performance (Sargent et al., 2020), and in this study, we investigated the usability of the beverage preparation machines and the impact on beverage preferences.

First, we looked at the comparison of both machine interfaces. Using the self-reported ratings and the behavioral performance (preparation time) measures, we were able to observe that the market-leading machine scored a higher rating, and participants were faster when preparing the beverage on this machine compared to the follower machine. While arousal measures did not indicate a significant difference during beverage preparation between the market leader and follower machine, frontal asymmetry results did during the “with instructions” block. Lower scores seen in frontal asymmetry indicate more relative left frontal activity which is associated with more positive emotional valence (Coan and Allen, 2004). A higher score indicated more relative right frontal activity which is associated with negative emotional valence (Coan and Allen, 2004; Palmiero and Piccardi, 2017). The follower machine showed more left frontal activity which indicates an approach motivation or more positive valence (Harmon-Jones and Allen, 1997, 1998; Allen et al., 2001; Coan et al., 2001; Harmon-Jones, 2003). This could indicate that the follower machine in the presence of instructions made participants more comfortable using it. Behavioral results showed the participants took longer to prepare the beverage on the follower machine. It suggested that the operation of this machine was more complicated than that for the market leader. The higher left cortical activity for the follower machine with instructions could also support this that when they had instructions, they were more at ease using the machine. The follower did show higher right frontal activity during the no instructions block which could indicate that without instructions the machine was more difficult to use and therefore causing more of a withdrawal motivation or negative emotional valence towards the machine. Research shows that when tasks become too complicated, consumers are no longer willing to perform them on their own and would rather have someone perform the task for them or not do it at all (Wolf and Mcquitty, 2011). The leader machine, on the other hand, did not show a significant difference between either block indicating that participants were either comfortable using the machine or did not have trouble with making the hot beverage and therefore their valence towards the machine remained similar in both conditions.

Next, we looked at the effect of the machine interface on preference for the prepared product. While we did not observe a significant difference in self-reported beverage likeability and behavioral performance, the product consumption measures and the brain and body measures were able to provide a more nuanced understanding. During beverage consumption, higher arousal and left-frontal activity for the leader machine indicated an approach motivation or more positive valence leading to higher preference for the beverage prepared on that machine during the free choice condition (Allen et al., 2001; Coan et al., 2001; Harmon-Jones, 2003). During the constrained choice, there was no significant difference between the beverage produced on either machine. Therefore, we conclude that the leader machine led to a higher product preference as measured by arousal and valence during consumption. In other words, when a hot beverage machine interface is easy and simple to use, consumers place a higher valuation on the beverage measured through arousal and valence. These findings are consistent with past research which suggests that consumers perceive a product to be of a higher value if they themselves prepare that product (Xie et al., 2007; Wolf and Mcquitty, 2011; Atakan et al., 2014). This result is also consistent with the idea that an optimal and efficient user interface impacts user attitudes towards the product (Hubert and Kenning, 2008).

This study also demonstrated the applicability of a multimodal neuroergonomic approach to marketing research by being able to study the brain and physiological responses while the participant was mobile and directly interacting with the machines and preparing and consuming the products in a naturalistic setting (Parasuraman and Wilson, 2008; Mehta and Parasuraman, 2013; Ayaz and Dehais, 2019; Rahman et al., 2019). Our multimodal approach proposes a more comprehensive way to measure consumer preference for products beyond that offered by traditional surveys. We were able to provide a deeper understanding of the user’s product preference and depict a more accurate description of their affective state. Finally, these results also support that utilizing wearable sensors can unobtrusively and continuously monitor a participant without interrupting the task that self-reported measures often require. One limitation is due to the mobile nature of the study we were unable to get clear signals from F3/F4 electrode pairs which are also typically used in calculating frontal asymmetry (Davidson et al., 1979, 1990). Further studies where both F3/F4 and F7/F8 channel pairs can be compared could further confirm the results of this study. Also, due to the exploratory nature of the study, a larger sample size could further confirm our results and also show a more significant difference between user interfaces.

These results not only enhance our understanding of consumers’ interaction with simple devices like a single-serve coffee machine but also can help the design of new devices. By exploring arousal from EDA and frontal asymmetry from EEG we can objectively confirm user preference for the leader machine. We did not confirm our expectation that the market leader machine would have a more efficient user interface than a market follower. However, we did show that the leader machine did have a higher product preference for the beverage produced in all results. This is the first study that uses a multimodal and comprehensive assessment of coffee machine use and consumption in a naturalistic work environment and provides a novel experimental design for conducting marketing research involving products. Approaches described here can be adapted in the future to optimize everyday tools and their usability.

## Data Availability Statement

The datasets generated for this study are available on request to the corresponding author.

## Ethics Statement

This study was carried out in accordance with the recommendations of Drexel University Institutional Review Board (IRB) with written informed consent from all subjects. All subjects gave written informed consent in accordance with the Declaration of Helsinki. The protocol was approved by the Drexel University IRB.

## Author Contributions

HA and RS initiated and supervised the project. AS and JW carried out the experiment. AS, JW, and HY processed and analyzed the data. All authors discussed and interpreted the results. AS wrote the manuscript with support from JW. HA and RS provided critical revisions to the manuscript.

## Conflict of Interest

The authors declare that the research was conducted in the absence of any commercial or financial relationships that could be construed as a potential conflict of interest.
